# Mibianto: ultra-efficient online microbiome analysis through *k*-mer based metagenomics

**DOI:** 10.1093/nar/gkae364

**Published:** 2024-05-08

**Authors:** Pascal Hirsch, Leidy-Alejandra G Molano, Annika Engel, Jens Zentgraf, Sven Rahmann, Matthias Hannig, Rolf Müller, Fabian Kern, Andreas Keller, Georges P Schmartz

**Affiliations:** Chair for Clinical Bioinformatics, Saarland University, 66123 Saarbrücken, Germany; Chair for Clinical Bioinformatics, Saarland University, 66123 Saarbrücken, Germany; Chair for Clinical Bioinformatics, Saarland University, 66123 Saarbrücken, Germany; Algorithmic Bioinformatics, Center for Bioinformatics Saar and Saarland University, Saarland Informatics Campus, 66123 Saarbrücken, Germany; Saarbrücken Graduate School of Computer Science, Saarland Informatics Campus, 66123 Saarbrücken, Germany; Algorithmic Bioinformatics, Center for Bioinformatics Saar and Saarland University, Saarland Informatics Campus, 66123 Saarbrücken, Germany; Clinic of Operative Dentistry, Periodontology and Preventive Dentistry, Saarland University Hospital, Saarland University, Kirrberger Str. 100, Building 73, 66421 Homburg, Saar, Germany; Helmholtz Institute for Pharmaceutical Research Saarland (HIPS), Helmholtz Centre for Infection Research, 66123 Saarbrücken, Germany; Deutsches Zentrum für Infektionsforschung (DZIF), Standort Hannover-Braunschweig, 38124 Braunschweig, Germany; PharmaScienceHub, 66123 Saarbrücken, Germany; Chair for Clinical Bioinformatics, Saarland University, 66123 Saarbrücken, Germany; Helmholtz Institute for Pharmaceutical Research Saarland (HIPS), Helmholtz Centre for Infection Research, 66123 Saarbrücken, Germany; Chair for Clinical Bioinformatics, Saarland University, 66123 Saarbrücken, Germany; Helmholtz Institute for Pharmaceutical Research Saarland (HIPS), Helmholtz Centre for Infection Research, 66123 Saarbrücken, Germany; PharmaScienceHub, 66123 Saarbrücken, Germany; Chair for Clinical Bioinformatics, Saarland University, 66123 Saarbrücken, Germany

## Abstract

Quantifying microbiome species and composition from metagenomic assays is often challenging due to its time-consuming nature and computational complexity. In Bioinformatics, *k*-mer-based approaches were long established to expedite the analysis of large sequencing data and are now widely used to annotate metagenomic data. We make use of *k*-mer counting techniques for efficient and accurate compositional analysis of microbiota from whole metagenome sequencing. Mibianto solves this problem by operating directly on read files, without manual preprocessing or complete data exchange. It handles diverse sequencing platforms, including short single-end, paired-end, and long read technologies. Our sketch-based workflow significantly reduces the data volume transferred from the user to the server (up to 99.59% size reduction) to subsequently perform taxonomic profiling with enhanced efficiency and privacy. Mibianto offers functionality beyond *k*-mer quantification; it supports advanced community composition estimation, including diversity, ordination, and differential abundance analysis. Our tool aids in the standardization of computational workflows, thus supporting reproducibility of scientific sequencing studies. It is adaptable to small- and large-scale experimental designs and offers a user-friendly interface, thus making it an invaluable tool for both clinical and research-oriented metagenomic studies. Mibianto is freely available without the need for a login at: https://www.ccb.uni-saarland.de/mibianto.

## Graphical Abstract

**Graphical Abstract:**
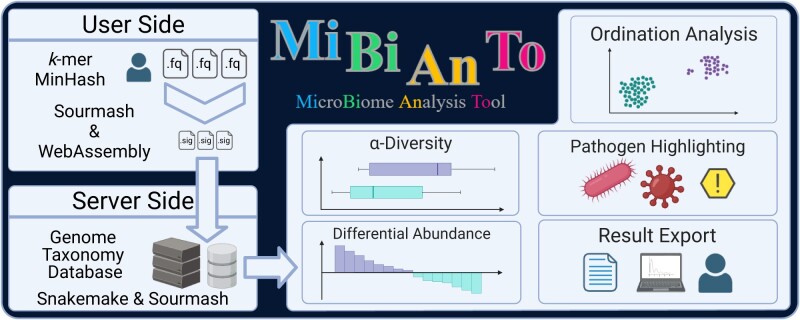

## Introduction

Technological advancements are rapidly transforming the research practices of molecular microbiology and ecology. Although the initial application of high-throughput sequencing focused on bacterial isolates only, further refinement of DNA preparation protocols has enabled the profiling of entire micro-ecosystems via metagenomics (1). If the challenges arising from whole metagenome sequencing data analysis can be overcome, likely unprecedented valuable insights can be gained into diverse research areas such as bioremediation, natural compound discovery, and human health (2–5). In practice however, whole metagenome sequencing experiments generate enormous quantities of sequencing data, rendering sufficient expertise in computational analysis indispensable (6). To support researchers in their data evaluation workflow, a wide variety of online data processing tools have emerged. MG-RAST (7), MGnify (8), Galaxy (9) and similar tools accept uploads of entire raw datasets and process them on their servers through custom data analysis pipelines. While this method of data management is intuitive and allows for in-depth analysis, it requires a fast internet connection on the user side, a solid infrastructure on the service provider side, and a considerable amount of processing time. For studies involving sensitive clinical data, users must consider all legal aspects before trusting any third parties. Furthermore, with continuously decreasing sequencing costs, most cohort studies steadily increase their data output, rendering large data exchange pipelines a major inconvenience for most end-users. One way to circumvent this issue is to request users to preprocess all raw data on their end before uploading it to a service that performs the downstream analysis. For instance, our tool BusyBee Web provides comprehensive binning functionality at the expense that users need to upload a complete assembly (10). In a similar manner, the tool MicrobiomeAnalyst, performs extensive downstream analysis on taxonomic counts (11), for which users are required to generate and upload a count matrix from the raw sequencing data. Even though many out-of-the-box solutions exist to solve both mentioned user-imposed challenges, lack of sufficient computational expertise prevents many researchers from accessing them. An alternative approach to addressing the challenges posed by the vast volumes of data in metagenomics is to leverage recent innovations in hashing-based data analysis techniques, exemplified by methods such as STAT (12) and Sourmash (13). These tools utilize efficient algorithms and approximations to handle large datasets effectively. Building upon these advancements, innovative web servers like PebbleScout (14) and Branchwater (15) have emerged, specializing in rapidly identifying the most similar samples within expansive databases.

Here, we propose Mibianto, an online whole metagenome sequencing data analysis web server centered around compositional analysis. It is light on user connection requirements, trivial to get started, and capable of quickly processing small to medium-sized studies. The tool leverages the recent progress made in FracMinHash-based data analysis to compute and transmit a compressed representation of the data to the remote server where it then performs taxonomic profiling (16). Once a job submission is finished, it provides a wide range of state-of-the-art analysis options, visualizations, and recommendations for further interactive analysis.

## Materials and methods

Mibianto is composed of three main components. First, the initial submission interface, where users are prompted to select metagenomic reads and metadata. Second, our server-side data analysis pipeline, which handles most of the computationally intensive tasks. Lastly, the result interface offers interactive data exploration, sharing and exporting capabilities.

### Taxonomic profiling

Mibianto circumvents transferring sequencing reads directly. Instead, it transfers a small sketch of *k*-mers sampled from the reads using the FracMinHash (16,17) implementation of the WebAssembly version of Sourmash (13). A *k*-mer is a short sequence of fixed length $k$ (using $k = 51$ in this concrete case). The set $W$ of all *k*-mers contained within a FASTQ file is reduced to a small sample of average fraction $1/s$ for a scaling constant $s >1$ as follows. Given a hash function h that maps each of the ${{4}^k}$ possible DNA *k*-mers to an integer i in the interval $[ {0,\ H} ]$, we only select *k*-mers whose hash value is at most $H/s$, i.e. from $W$, we keep only the sketch of integer hash values


\begin{eqnarray*}{\mathrm{FRA}}{{{\mathrm{C}}}_{s\left( W \right)}} := \left\{ {h\left( W \right)\; | w \in {\mathrm{W\ for\ which\ h}}\left( {\mathrm{w}} \right) \le \frac{H}{s}} \right\}.\end{eqnarray*}

Note that the size of this set varies for each sample but is on average $1/s$ of the original size of $W$. These sketches can be used to estimate overlap (Jaccard coefficients) or containment between large sets while using only a small fraction of the space. To be able to perform this computation on the client side, sourmash was compiled from Rust to WebAssembly. We achieved this conversion with the rust package manager cargo (v:1.65.0), rustc (v:1.65.0), wasm-pack (v:0.9.1), and sourmash (v:4.6.1). Via JavaScript the user input files get decompressed and streamed to the WebAssembly package after which it is transmitted to the server. For reference, the computation of a 150 bp paired-end sample containing 8 Gbp takes around 15 minutes on a standard consumer laptop. Users may select to save their metadata and hashes on the web server for later reusage. Once the data upload is completed, taxonomic profiling starts, and the user may enter a waiting queue and receive a unique job identifier. To ease software maintenance, we implemented the taxonomic profiling pipeline in snakemake (v:7.18.2) (18). Data processing closely follows the sourmash documentation from GitHub at a fixed *k*-mer size of 51. Sketches of each sample are compared against the Genome Taxonomy Database (GTDB) (v:rs207) (19) with the sourmash gather command. Based on this these results we approximate taxonomic abundances following the proposed methodology of Chou and Reiter (20). Next, taxonomic counts of all samples are aggregated, taxonomic annotations, sample data, and a phylogenetic tree are attached, and a phyloseq (v: 1.42.0) object is saved (21). Upon successful completion of the server-side processing pipeline, the user is forwarded from the queue to the results page, where a wide range of further analyses can be performed, and two download options are available. A phyloseq object and an Excel file with taxonomic counts can be downloaded, aiming to serve users with and without programming skills, respectively.

### Compositional analysis

Users that prefer additional support can refer to our results page for state-of-the-art compositional analyses supported with rich interactive visualization and customization options. We also implemented various data normalization options, namely compositional, *z*-score, log_10_, log_10_*p*, hellinger, centered log-ratio and additive log-ratio. Moreover, users may filter their data by removing individual samples or operational taxonomic units (OTU) based on abundance criteria. Alpha diversity can be visualized and checked for significant differences among cohort or sample groups. Further, ordination analysis is supported by a range of dimensionality reduction methods and dissimilarity measures. To this end, we integrated major parts of microViz, which allows for an interactive selection of samples in the embedding and displays their relative composition (22). We also provide a table view where estimated abundances are numerically displayed for each taxonomy. Individual OTUs can be selected, and their normalized abundances are displayed across samples. Most relevant for clinical applications, we use a list of potentially pathogenic species from gcPathogen (23) to automatically highlight them among the results. In case higher taxonomic ranks are of interest, we highlight an OTU as a potential pathogen when it contains at least one potentially pathogenic species. Finally, we implemented differential abundance analysis with ANCOMBC (v:2.0.1) (24).

### Proxies for quality control

Quality control (QC) is a crucial step of every sequencing analysis workflow. Performing QC on the user side would aggravate the computational burden on their end. However, since Mibianto only transfers hashed information to the server, QC on the server side becomes a challenge. We addressed this issue by computing several proxies for QC and forwarding users to further online analysis with e.g. BusyBee Web in case of apparent data anomalies. First, the estimate of the overall assignment rate from sourmash is displayed to the user on the results page indicating how robustly the community of each sample was quantified. Low assignment rates indicate either that the submitted community is not adequately represented in the database or that quality issues exist. Second, we compare user hashes against a precomputed QC dataset by computing distances with the built-in comparison from sourmash. This QC dataset was compiled by downloading metagenomics samples along with annotations from SRA. Adapter and host DNA contaminations were estimated with trimgalore (v: 0.6.10) (25) and bowtie2 (v: 2.5.1) (26) was used for the alignment against the human genome. Spatial proximity in co-embeddings of QC samples with increased contamination might be indicative of similar issues in user data. For clinical cohort studies, we annotate potential outliers using the local outlier factor (LOF) from DescTools (v: 0.99.47) (27). We note that the local outlier factor highlights abnormal clustering behavior of individual data points. Yet, depending on the experimental setup, this may be expected.

### Case studies

To explore the potential and limitations of our new online tool, we provide an example analysis of two different datasets, comprising one classical cohort study, seven biospecimens, four DNA extraction protocols, and two different sequencing technologies. The datasets by Rehner *et al.* (28) and Becker *et al.* (29) were fully processed with our implemented snakemake pipeline to ensure that results can be replicated. Original metadata classes were curated. Pipeline outputs were integrated into the web server and explored using the results page. In the cohort study, the 4-week timepoint was removed. The dataset by Rehner *et al.* (28) reports DNA sequencing obtained from bile, stool, saliva, plaque, sputum, conjunctiva, and water control samples with the Qiagen DNeasy PowerSoil Pro (QPS), Qiagen QiAamp DNA Microbiome Kit (QMK) and ZymoBIOMICS DNA Miniprep Kit (ZYMO). For each biospecimen, all aliquots were derived from the same biological sample. Additionally, to the short read MGI sequencing datasets, matched Oxford nanopore sequencing is available for saliva and bile samples. Full details on experimental design may be found in the corresponding manuscript (28). The dataset by Becker et al. (29) consists of 140 stool samples and three cohorts, namely Parkinson's disease (PD), Parkinson's Disease with a resistant starch intervention (PD + RS), and the control cohort. The PD cohort received dietary instructions, whereas the control and PD + RS cohorts received resistant starch as a nutritional supplement. Measurements were taken at different timepoints. Full details may be found in the respective manuscript (29).

## Results and discussion

The impact of the human microbiome on host health as mediated by various exogenous molecules like complex peptides and metabolites is well perceived (30,31). While extensive research is improving our understanding of the causal mechanisms linking microbiota and the immune system, disease associations with microbiome composition can provide valuable insight for clinicians (32,33). However, quantifying microbiome composition from metagenomic sequencing reads remains time-consuming and computationally challenging. Recent developments in the field of sketch-based taxonomic profiling have resulted in an efficient solution for exchanging large quantities of metagenomic sequencing data between client and server. By leveraging sourmash as a taxonomic profiling backbone, we have developed Mibianto, an online solution for convenient microbiome composition analysis. We provide a wide range of state-of-the-art downstream analyses with many customization options, partially based on the microViz package. The functionality includes assessment of different taxonomic ranks, data filtering, diversity analysis, pathogen highlighting, and more. We are aware of the workflow's QC limitations and have therefore provided several indicators to detect potentially contaminated samples.

### Mibianto handles metagenomes from saliva, skin, plaque, stool and eye samples

We aimed to rigorously evaluate the performance of Mibianto across diverse experimental setups. A critical factor influencing metagenomic outcomes is the type of sample being analyzed. Metagenomes derived from saliva, gut, dental plaque, skin, or the eye exhibit significant variations in microbial composition, reflecting the unique microbiota habitats of these biological sources. Moreover, the choice of DNA extraction protocols can greatly impact the variability of results. Different methodologies may preferentially extract certain microbial groups, leading to variation in observed community structures. Therefore, comprehensive testing across these varying conditions is crucial to ensure tool robustness and reliability in accurately capturing and reflecting the intricate diversity and dynamics of microbial communities. In the first dataset by Rehner et al. we thus compare three different DNA extraction kits on seven different biospecimens, compiling a total of 30 data points (28).

**Figure 1.**
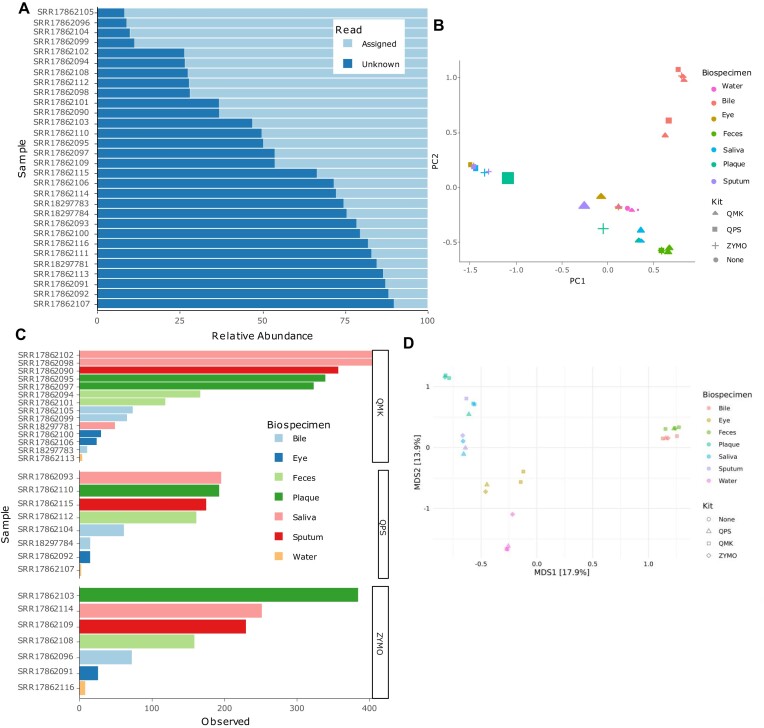
Mibianto results of the protocol comparison after minor adjustments to the visualizations downloaded from the server. (**A**) Assignment rate for all samples. Samples with the prefix SRR18 were sequenced with Oxford nanopore sequencing. (**B**) Quality control proxy computed on FracMinHash-based dissimilarities without co-embedding of our precomputed samples. (**C**) Number of observed species in each sample, split by DNA extraction kit. (**D**) Short-read sequencing samples were embedded with principal coordinate analysis on Bray-Curtis distances computed on the species level.

The assignment rates displayed by Mibianto are decreased for the almost sterile samples of water and conjunctiva already suggesting lower quality of the samples (Figure 1A). Further, in saliva and bile samples, nanopore reads have lower assignment rates compared to their short-read counterpart. The FracMinHash-embedding without Mibianto's internal data indicated QPS sputum as an outlier (Figure 1B). We note that the samples we describe here were already human-read decontaminated by Rehner et al., accordingly the samples did not cluster with our selection of highly contaminated samples. Partially reconstructing the original analysis from the manuscript with Mibianto on a species level, the highest number of observed species is found in the oral cavity in QMK and ZYMO, which corroborates the findings of the original manuscript (Figure 1C). Water contamination is highest with ZYMO. Principal coordinate analysis on Bray-Curtis distances of short reads clusters samples by biospecimen (Figure 1D). Bile and stool cluster closely together. Following the decision in the original manuscript, we do not perform differential abundance analysis due to the high number of confounding variables and lack of replicates.

### Mibianto identifies significantly de-regulated gut microbiome species in Parkinson's disease

After testing its robust performance across various species types and DNA extraction methods, we next evaluate Mibianto's efficacy in analysing case–control metagenomic studies. It is designed to facilitate the dissection of microbial variations between control and case groups, offering valuable insights into microbial dynamics. In that aspect, we aim to position Mibianto as a powerful tool for medical and life-scientific researchers with an interest in understanding differences within microbiomes across diverse research settings. The second dataset is a next-generation sequencing dataset of an interventional cohort study on Parkinson's disease by Becker *et al.* (29).

**Figure 2.**
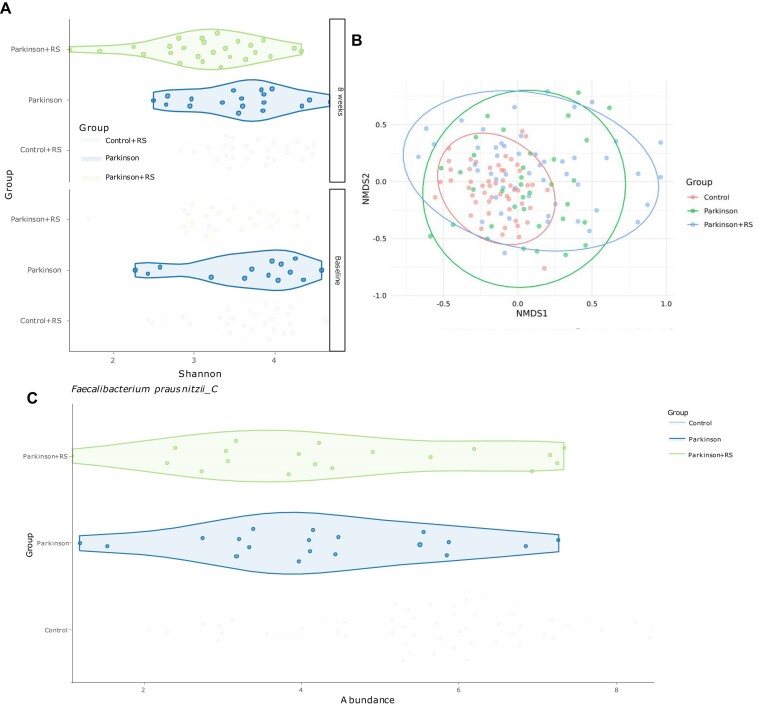
Mibianto results of the cohort study after minor adjustments to the visualizations downloaded from the server. (**A**) Shannon diversity computed on species level grouped by cohort and split by timepoint. (**B**) Ordination analysis using non-metric multidimensional scaling on Bray-Curtis distances. (**C**) Abundance of F. *prausnitzii_C* in the different cohort groups after center log-ratio normalization.

Data exploration with Mibianto on the species level indicates a high assignment rate with a few outliers. We observed statistically significant differences in alpha diversity before adjustment comparing PD + RS against PD (Wilcoxon Mann–Whitney *p*-value ≈ 0.0306) and the control (*p*-value ≈ 0.0002), yet no significant difference was observed between PD and control (*p*-value ≈ 0.3185) (Figure 2A). Ordination analysis clusters control samples visibly closer together (Figure 2B). Differential abundance analysis with ANCOMBC and Benjamini–Hochberg *p*-value adjustment highlighted 33 and 17 OTUs as significant differentially abundant among controls and PD + RS as well as controls and PD, respectively. No significant differences were detected comparing PD with PD + RS. We want to emphasize that the significance of all *p*-values mentioned in this section is artificially inflated, because the samples are not statistically independent due to the aggregation across timepoints. In both contrasts, the most significant differentially abundant OTU was *Faecalibacterium prausnitzii_C* (Figure 2C). *F. prausnitzii* is described to have anti-inflammatory effects and is known to be depleted in Parkinson's disease patients (34,35). Further it can produce butyrate, a short chain fatty acid, whose decreased concentration has been associated with depression in Parkinson's disease (36). By contrast, increased levels have been shown to decrease motor deficits in the animal model (37).

### Mibianto compresses metagenomic data sets by a factor of up to 245

An important aspect of Mibianto is to facilitate the online analysis of larger studies by reducing the transferred data set at the client side. From the previous studies we estimate how many bytes are transferred from the user site to the server site of Mibianto, after the *k*-mer spectra are generated on the local computer. The data compression ratio was about 245:1, resulting in a data size reduction of 99.59%. This enables the user to analyze big data sets with minimal data upload.

### Enabling specific analyses using BusyBee Web

While Mibianto excels at managing large-scale studies through efficient data compression, it is recognized that certain in-depth analyses fall outside the scope. To address this, we have seamlessly integrated Mibianto with our previously developed BusyBee Web platform. BusyBee Web is tailored for a distinct purpose: conducting extensive in-depth analyses of a smaller number of metagenomic samples. Unlike Mibianto, which operates on compressed data, BusyBee Web requires a full upload of metagenomic datasets. This complementary approach allows Mibianto to identify and propose a subset of samples that warrant more detailed examination. Leveraging both platforms in tandem enables researchers to navigate from broader metagenomic surveys to focused, in-depth analyses with ease and precision. To enable this feature, we automatically display a list of ten samples with the overall highest normalized abundance of potential pathogens to hint at another in-depth analysis with BusyBee Web.

### Outlook: ultra-high processing performance for eukaryotic reads

We evaluate the possibility to extend the concept of Mibianto—namely to use *k*-mer spectra with a reduced size for performing web-based analyses at client side – to human nucleic acid data sets. As one of the most frequent use-cases we considered gene expression profiling. To test this, we adapt the fast gapped *k*-mer counter hackgap (38), which is based on a 3-way bucketed cuckooo hash table (39). We only index *k*-mers that occur in a single gene, considering all transcripts of the gene. To process an RNA-seq sample, we count the occurrences of each indexed *k*-mer in the sample. A robust average (trimmed mean) of the *k*-mer counts is a measure of the gene expression, if sufficient care is taken to account for inflated zero counts from *k*-mers belonging to transcripts that are not expressed at all in the sample.

**Figure 3.**
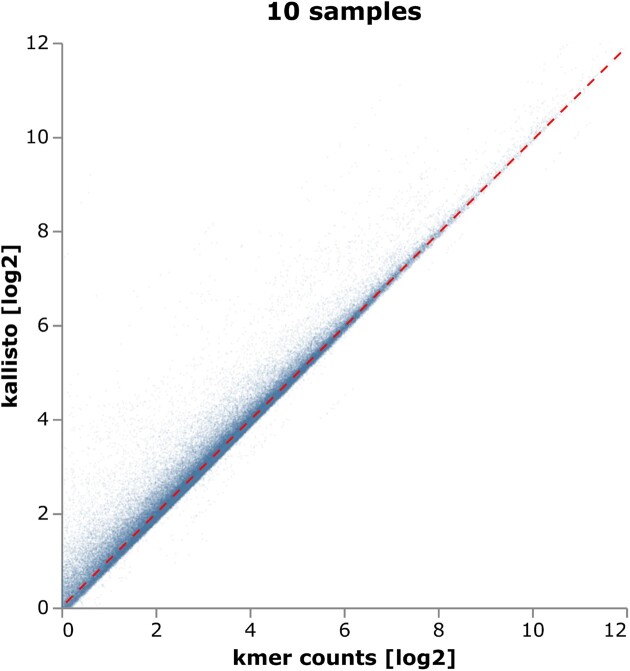
Scatter plot of normalized base-2 logarithms of *k*-mer counts vs. kallisto FPKM values; one point per expressed gene per sample from 10 different samples (103.415 points overall), with a trend line (dashed red) obtained by robust regression. Partial transparency was used to visualize regions of low vs. high point density. Overall, a strong correlation is visible (Pearson correlation coefficient 0.983).

To evaluate whether *k*-mer count based expression values agree with established measures of expression (here, FPKM values computed by kallisto (40)), we arbitrarily chose 10 publicly available RNA-seq samples from the Gene Expression Omnibus project ID GSE79362 (SRR3235783-87, 89–90, 92–94) (41) and calculated gene expression measures based on both our robustly averaged *k*-mer counts and the standard kallisto FPKM values. Our results show overall a very good agreement on the reliably expressed genes (average *k*-mer count ≥ 1, at least 500 *k*-mer counts available) with a Pearson correlation coefficient of 0.983, and a slope of 0.99 for the regression line (Figure 3).

In the future, we intend to reduce the *k*-mer set to a minimal but representatively samples *k*-mer spectrum per gene. We aim to reliably estimate human gene expression from a small fraction of the initially available information, like Mibianto already accomplishes in the case of metagenomic data.

## Conclusion

Mibianto is a web server that specializes in the compositional data analysis of metagenomic sequencing data. It distinguishes itself from existing online solutions by input flexibility, ease of use, and minimal connection requirements. This aspect is particularly beneficial for large-scale case-control studies, as the web server streamlines the processing of hashed sequencing data and emphasizes pathogen identification. However, incorporating functional analysis, de-novo assembly, genome mining, or any analysis requiring access to larger pieces of DNA sequence is currently not possible due to the specific design of our client-server data exchange model. Additionally, further research is required in the field of FracMinHash-based taxonomic profiling to refine results for nanopore sequencing reads (42). Nonetheless, Mibianto already provides a wide range of features and functionalities that enable rapid insights into microbial communities with extensive result visualization and the ability to customize to individual workflows. Based on two previously published datasets, we demonstrated how our tool was able to confirm central findings in metagenomic experiments without the need for any deeper bioinformatics expertise. Mibianto was designed to serve as a valuable tool for researchers in metagenomics and related fields and we invite users to suggest additional desired features or ideas on our GitHub project page (https://github.com/CCB-SB/mibianto).

## Data Availability

Mibianto is freely available without any login requirement at: https://www.ccb.uni-saarland.de/mibianto.

## Accession numbers

Data for the cohort study was made available by Becker *et al.* (29) on NGDC GSA under the accession: HRA000635.

Data for the protocol study was made available by Rehner *et al.* (28) on NCBI SRA under the accession: PRJNA802336.

The data used for the QC dataset was compiled by using following metagenomics samples along with their annotations from SRA: SRX14313109, SRX14313060, SRX14864825, SRX14864839, SRX14864822, SRX14648162, SRX19039438, SRX16004454, SRX16004468, SRX16004467, SRX15455164.
